# Pure Androgen-Secreting Radiologically Suspicious Adrenal Mass: Benign or Malignant?

**DOI:** 10.7759/cureus.26234

**Published:** 2022-06-23

**Authors:** Chaitra Gopinath, Suman Shekar, Madan Acharya, Vishwanath Pattan, Vishnu Sundaresh

**Affiliations:** 1 Diabetes and Endocrinology, Kansas City University, Kansas City, USA; 2 Medical Center Primary Care, University of Kentucky Bowling Green Campus, Bowling Green, USA; 3 Cardiology, Unity Point Health, Des Moines, USA; 4 Diabetes and Endocrinology, Upstate University Hospital, Syracuse, USA; 5 Division of Endocrinology, University of Utah School of Medicine, Salt Lake City, USA

**Keywords:** hyperandrogenism, dhea-s, testosterone, adrenocortical tumors, adrenal mass

## Abstract

Pure androgen-secreting adrenocortical tumors (PASATs) are rare entities. Their clinical presentations include virilizing features that vary based on age and gender. The pathogenesis of this tumor is still unclear, with around 50% of such tumors being malignant. Imaging characteristics of the tumor on CT/MRI including size, heterogenicity, and contrast wash-out time are used to predict malignancy. Surgical excision is recommended for all functional adrenal tumors. In this report, we discuss a case of a 68-year-old postmenopausal female presenting with hyperandrogenism and was found to have a 7-cm, PASAT that raised suspicion for malignancy on CT scan, but was determined to be benign on surgical pathology.

## Introduction

Adrenal incidentalomas are estimated to be present in about 2% of the population, with a peak incidence between the fifth to seventh decades of life, and adrenocortical carcinomas make up about 2% of these cases [1]. Adrenocortical tumors that exclusively produce androgens are extremely rare. In a retrospective study of 470 adrenalectomies performed over a period of 56 years from January 1946 to November 2002 at the Mayo Clinic, 11 pure androgen-secreting tumors were reported [2]. These tumors are more common in females, and present with virilizing features of hirsutism, acne, deepening of the voice, and clitoral enlargement [2,3]. Due to their extremely low prevalence, there is a paucity of data on the risk of malignancy for pure androgen-secreting adrenal tumors (PASATs). CT or MRI characteristics are used to predict the malignancy of adrenal tumors in general. Predictors of benign adenoma include size <4 cm, <10 Hounsfield units (HU) on unenhanced CT, or contrast wash-out >50% at 10 minutes [4]. A fluorine-18 fluorodeoxyglucose positron emission tomography-CT (18F FDG PET-CT) has a 91% sensitivity and specificity but is expensive and not widely available [1]. In addition, 24-hour urine steroid profiling is a recent methodology to differentiate between benign and malignant adrenal tumors [5]. We present a 68-year-old female patient with hirsutism and a 7-cm adrenal nodule suspicious for malignancy based on the size and imaging characteristics but was proven otherwise on surgical pathology.

## Case presentation

A 68-year-old postmenopausal female presented with excess facial hair around the chin for 18 months. She did not have excess hair in other areas of her body. She endorsed thinning of hair on the crown of her head. She did not report a change in her voice. She had no prior history of polycystic ovarian syndrome or long-term glucocorticoid use. At the age of 51 years, she had breast cancer managed with lumpectomy followed by radiation therapy and had no evidence of recurrence on follow-up imaging. At age 52, she had undergone a hysterectomy with bilateral oophorectomy for cervical intraepithelial neoplasia. She was not using any medications or supplements containing androgen. Her husband never used topical androgen replacement. She reported no family history of adrenal tumors or related genetic syndrome. Physical examination revealed a BMI of 22.3 kg/m^2^, blood pressure of 126/80 mmHg, a few coarse thickened hair on the chin, and clitoral enlargement. She did not have acne or a deepening of the voice. There were no physical examination findings suggestive of hypercholesterolemia, overt Cushing syndrome, or acromegaly.

Laboratory investigations revealed normal serum electrolytes, elevated testosterone, dehydroepiandrosterone sulfate (DHEA-S), and estradiol while luteinizing hormone (LH) and follicle-stimulating hormone (FSH) were on the lower side of the postmenopausal reference range (Table 1).

**Table 1:** Laboratory test results SHBG: sex hormone-binding globulin; DHEA-S: dehydroepiandrosterone sulfate; LH: luteinizing hormone; FSH: follicle-stimulating hormone; ACTH: adrenocorticotropic hormone

Laboratory findings	Preoperative	1 month postoperative	3 months postoperative	2 years postoperative	3 years postoperative	4 years postoperative	5 years postoperative	7 years postoperative
									Total testosterone (5-32 ng/dL)	110	7	5	4	4	5	4	9
Free testosterone (0.6-3.8 pg/mL)	18.2	1	0.8	0.5	0.5	0.7	0.6	1.4
SHBG (30-135 nmol/dL)	31	39	37	58	49	47	40	38
DHEA-S (13-130 µg/dL)	730	10	14	15	19	20	18	24
Androstenedione (0.130-0.820 ng/ml)	6.44			0.138	0.133	0.246	0.138	0.335
Estradiol (postmenopausal: <41 pg/mL)	65	<20		2.9	3.2			2.2
LH (postmenopausal: 7.7-58.5 IU/L)	20.5			30.2	30.8	30.8	27	29.4
FSH (postmenopausal: 25.8-134.8 IU/L)	27.9			55.4	56.3	55.8	54.9	54.9
Aldosterone (4.0-31.0 ng/dL)	15.4							
ACTH (7.2-63.3 pg/mL)		22		24	6	25.6	21.7	
Renin activity (0.5-4.0 ng/mL/hr)	1.6							
Plasma metanephrine (0.0-0.49 nmol/L)	0.13							
Plasma normetanephrine (0.0-0.89 nmol/L)	0.54							
24-hour urine free cortisol (<45 ug/L)	7.1		12	9.3	8.4	10.4	7.7	

**Figure 1:**
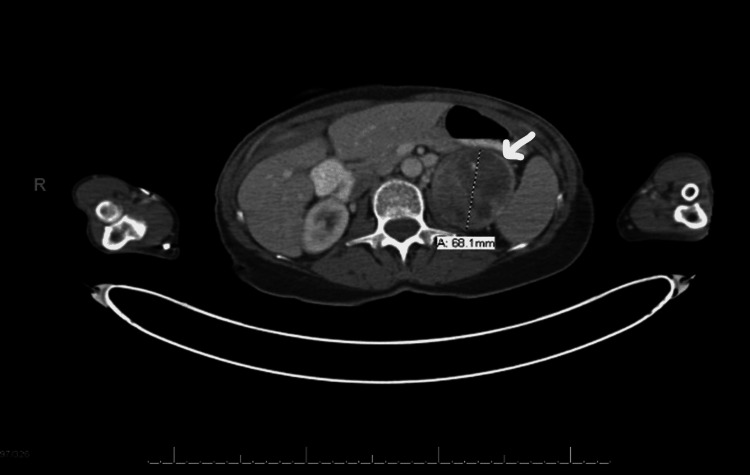
PET/CT image (preoperative) PET/CT: positron emission tomography/computed tomography

Plasma metanephrines, aldosterone, renin activity, and 24-hour urine free cortisol were within the reference range. An abdominal CT scan with adrenal protocol revealed a 7.0 x 7.3 x 7.5-cm left adrenal mass with heterogeneous enhancement with small areas of central calcification. There was no wash-out, rather “wash­-in” with HU increasing from 35 to 70 at eight minutes and no evidence of abdominal metastasis (Figure 1).

The adrenal mass had displaced the left kidney and pancreas without evidence of infiltration of surrounding structures. The 18F FDG PET/CT confirmed the above findings and did not reveal any abdominal metastasis.

**Figure 2:**
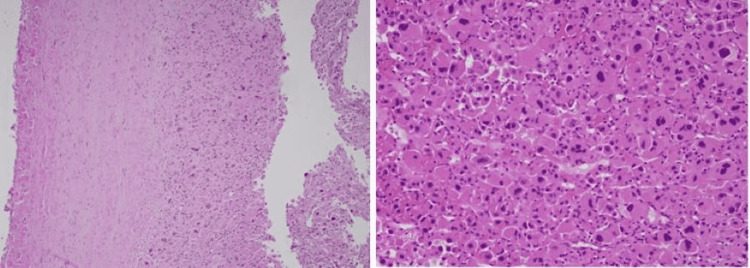
Surgical pathology of adrenal mass

The patient underwent laparoscopic left adrenalectomy. A 7-cm well-encapsulated adrenal tumor not adherent to any structures was visualized intraoperatively. Pathology showed a pleomorphic population of cells with rare mitotic figures, areas of hemorrhage, and fibrin deposition without necrosis surrounded by a thick fibrous capsule without lymphovascular or capsular invasion favoring a benign adenoma (Figure 2).

Postoperatively, the patient reported improvement in clinical symptoms. Levels of androgens and estrogen normalized and remained so seven years after surgery. Follow-up CT abdomen did not show any evidence of recurrence seven years postoperatively.

## Discussion

Features of hyperandrogenism in a postmenopausal female, as seen in our patient, is a diagnostic challenge and a malignant etiology is always a concern. Determining the source to be ovarian or adrenal is an important initial step. A thorough clinical history and physical examination in a postmenopausal woman are important in distinguishing the functional causes of hyperandrogenism from ovarian/adrenal tumors [6]. An absence of hyperandrogenic features during reproductive years, previous oophorectomy, normal BMI, and physical signs including an enlarged clitoris prompted us to consider an adrenal etiology as the most likely source of excess androgen in our patient.

The majority of the adrenal tumors are non-functional and are diagnosed incidentally on imaging. Excess hormone production is seen in about 10% of patients [1]. Adrenal carcinomas have an annual incidence of 0.7-2 cases per million per year [7]. Of these hormone-secreting tumors, PASATs are extremely rare, and hence only isolated case reports or case series have been published in the literature so far. A French study reported an incidence of 2.4% for PASATs among 801 adrenalectomies performed, and 50% were reported to be malignant [8]. These tumors can occur both in children and adults. In one study, the age at the time of diagnosis ranged from 1 to 53 years, and most studies report a higher incidence in females with a female-to-male ratio of 4:1 [2,9]. Clinical features of these tumors are representative of excess androgen production with hirsutism and virilization being present in 90-100% of the patients [2-3,9]. The pathogenesis of PASATs is largely unknown. Increased enzyme activity of 3β-hydroxysteroid dehydrogenase 2 and cytochrome P450 17 α-hydroxylase has been reported in a few cases [10].

DHEA-S is exclusively produced by the adrenal glands under the regulation of adrenocorticotropic hormone (ACTH). Testosterone, the most potent androgen, is produced by the adrenal cortex (25%) and ovarian stroma (25%). The remaining 50% is produced by the peripheral conversion from its precursors (androstenedione, DHEA, and DHEA-S). Measuring elevated levels of serum testosterone or DHEA-S has a sensitivity of 100% and specificity of 50% for detecting virilizing adrenal tumors [11]. A recent study demonstrated that a DHEA-S ratio (DHEA-S level divided by the lower limit of the DHEA-S reference range) ≤1.12 is also highly sensitive and specific for detecting autonomous cortisol production since the excess cortisol suppresses DHEA-S production [12]. The ratio in our case was greater than 1.0, suggesting an absence of autonomous cortisol production consistent with a 24-hour urine free cortisol on the lower side of the reference range at 7.1 ug/L (<45 ug/L). The androgens produced by virilizing adrenal tumors are generally unresponsive to ACTH stimulation due to the absence of ACTH receptors on the tumor cells [13].

Abdominal CT with adrenal protocol is the preferred initial method of imaging for adrenal glands in adults [1]. Szolar et al. (2005) analyzed CT characteristics of adrenal tumors and found that adrenocortical carcinomas were significantly larger than adenomas, with only 2.7% of adrenal carcinomas being less than 6 cm in diameter and had an unenhanced attenuation >10 HU [14]. A recent study by Park et al. showed that the sensitivity of adrenal CT protocols varies with size, and for tumors greater than 3 cm, many adrenocortical carcinomas cannot be distinguished from adenomas [15]. Moreover, there is a paucity of data on predictors of malignancy in PASATs. Cordera et al. (2003) reported that PASATs ranged in size from 1.5 to 6 cm, while the androgen-producing adrenal cancers ranged from 6 to 11 cm [2]. Thus, PASATs more than 6 cm could be considered suspicious for malignancy. Adrenal CT protocols include enhanced CT imaging 60 seconds after contrast administration and delayed enhanced images at 15 minutes. Early wash-in and wash-out of the contrast material on adrenal CT protocol is usually suggestive of benign pathology, with benign adenomas generally having an enhancement wash-out greater than 60% and relative enhancement above 40% [16].

Steroid metabolite profiling using either 24-hour urine or serum is another method that is currently being used to evaluate adrenal tumors. A prospective test validation study showed that urine metabolite measurement had a higher positive predictive value compared to tumor size or imaging characteristics [5]. Elevated levels of tetrahydro-11-deoxycortisol in a 24-hour urine sample are highly sensitive and specific for adrenocortical carcinoma [17]. However, urinary steroid profiling is cumbersome and expensive.

A biopsy is not recommended in an adrenal tumor suspicious for cancer due to poor diagnostic accuracy and the risk of needle track seeding [18]. Surgery for an adrenal tumor is indicated for size >4 cm and irrespective of the size if it is biochemically active. Laparoscopic adrenalectomy is the gold standard for adrenal tumors [19]. Our patient underwent laparoscopic adrenalectomy, which revealed a 7-cm well-encapsulated adrenal tumor. Modified Weiss criteria, which is based on histologic features, are one of the methods used to identify adrenocortical carcinomas. They include mitotic rate, atypical mitosis, necrosis, clear cells, and capsular invasion. A score of three or more is indicative of a carcinoma. Ki-67 index, β‐catenin, and synaptophysin are some of the immunohistochemical markers used mainly to differentiate adrenocortical carcinomas from pheochromocytoma and adrenal metastases [20]. Surgical pathology in our patient revealed a pleomorphic population of cells with rare mitotic figures, areas of hemorrhage, and fibrin deposition without necrosis and surrounded by a thick fibrous capsule without lymphovascular or capsular invasion. These findings were indicative of a benign adenoma. Subsequent follow-up showed normal androgen levels and no evidence of recurrence on CT scan seven years postoperatively.

## Conclusions

A malignant etiology is always a concern when postmenopausal patients present with clinical hyperandrogenism. Once biochemical hyperandrogenism is confirmed, imaging of the abdomen/pelvis leads us to either an adrenal or ovarian etiology. Large adrenal tumors with suspicious radiological features are considered malignant unless proven otherwise. It is challenging to differentiate benign PASATs from malignant ones based on imaging characteristics alone. A combination of imaging features and serum or urine steroid profile should be utilized to better characterize PASATs preoperatively, which will help guide surgical management. Our 68-year-old postmenopausal female patient presented with virilization and biochemical hyperandrogenism suggestive of a PASAT and a 7-cm adrenal mass with radiological characteristics suggestive of malignancy, which was surprisingly found to be benign on surgical pathology. This case is an exception to the general clinical rule for adrenal malignancy based on the size and radiological features.
